# Characterization of mammary epithelial stem/progenitor cells and their changes with aging in common marmosets

**DOI:** 10.1038/srep32190

**Published:** 2016-08-25

**Authors:** Anqi Wu, Qiaoxiang Dong, Hui Gao, Yuanshuo Shi, Yuanhong Chen, Fuchuang Zhang, Abhik Bandyopadhyay, Danhan Wang, Karla M. Gorena, Changjiang Huang, Suzette Tardif, Peter W. Nathanielsz, Lu-Zhe Sun

**Affiliations:** 1 Institute of Environmental Safety and Human Health, School of Laboratory Medicine and Life Science, Wenzhou Medical University, University Town, Wenzhou 325035, China; 2 Department of Cellular & Structural Biology, University of Texas Health Science Center, San Antonio, Texas 78229, United States; 3 Cancer Therapy and Research Center, University of Texas Health Science Center, San Antonio, Texas 78229, United States; 4 Flow Cytometry Facility, University of Texas Health Science Center, San Antonio, Texas 78229, United States; 5 Barshop Institute for Longevity and Aging Studies, University of Texas Health Science Center, San Antonio, Texas 78229, United States; 6 Southwest National Primate Research Center, Texas Biomedical Research Institute, San Antonio, TX, 78245, United States; 7 Center for Pregnancy and Newborn Research, University of Texas Health Science Center, San Antonio, Texas 78229, United States; 8 The Second Affiliated Hospital, Wenzhou Medical University, Wenzhou 325035, China

## Abstract

Age is the number one risk factor for breast cancer, yet the underlying mechanisms are unexplored. Age-associated mammary stem cell (MaSC) dysfunction is thought to play an important role in breast cancer carcinogenesis. Non-human primates with their close phylogenetic relationship to humans provide a powerful model system to study the effects of aging on human MaSC. In particular, the common marmoset monkey (*Callithrix jacchus*) with a relatively short life span is an ideal model for aging research. In the present study, we characterized for the first time the mammary epithelial stem/progenitor cells in the common marmoset. The MaSC-enriched cells formed four major types of morphologically distinct colonies when cultured on plates pre-seeded with irradiated NIH3T3 fibroblasts, and were also capable of forming mammospheres in suspension culture and subsequent formation of 3D organoids in Matrigel culture. Most importantly, these 3D organoids were found to contain stem/progenitor cells that can undergo self-renewal and multi-lineage differentiation both *in vitro* and *in vivo*. We also observed a significant decrease of luminal-restricted progenitors with age. Our findings demonstrate that common marmoset mammary stem/progenitor cells can be isolated and quantified with established *in vitro* and *in vivo* assays used for mouse and human studies.

Human population aging is accelerating in both the developing and the developed world^1^, and aging is a major risk factor for many types of malignancies including breast cancer. For example, women older than 50 years account for 80% of new breast cancer diagnoses^2^. However, the underlying reason for this age-associated incidence of breast cancer is unknown. More recently, stem cell exhaustion has been demonstrated as one of the hallmarks of age-related diseases^3^ in various tissues including the hematopoietic^4^,^5^,^6^, nervous^7^,^8^,^9^,^10^, gastrointestinal^11^, muscle^12^,^13^ and skin tissues^14^. In the human mammary gland, stem/progenitor cells have also been implicated to play an important role in breast cancer initiation^15^,^16^. A recent study indicated that dysfunctional mammary epithelial progenitor and luminal cells with acquired basal cell properties accumulate during aging^17^. However, whether altered stem/progenitor cell function is a major underlying cause for the increased incidence of breast cancer with aging is unexplored.

Though the rodent model has been extensively used for human breast cancer research and mammary stem cell research in the past, there are a number of significant differences between mammary glands in rodents and humans^18^. For example, the mouse mammary gland is composed of a linear ductal branching system with very little fibrous connective tissue around the ducts. Also the terminal end buds usually do not develop into alveolar structures except for during pregnancy. In contrast, the human mammary gland is composed of 11~48 central ducts that radiate outward from the nipple^19^. The human breast also contains much highly fibrous connective tissue surrounding the epithelial ducts and lobules. These distinct structural and compositional differences may in large part explain why spontaneous mammary tumors in mice do not resemble those found in humans^20^.

Direct study of human breast tissue to evaluate age-associated mammary stem cell (MaSC) functional changes is greatly limited by the lack of an adequate supply of normal human breast tissue across the life span. Alternatively, nonhuman primates, with their close phylogenetic relationship to humans, could prove an important resource to determine the effect of age on MaSCs. In particular, the common marmoset (*Callithrix jacchus*), one of the smallest anthropoid primates, has been proposed as a powerful model for aging research due to its relative short life span compared to other non-human primates such as the rhesus monkeys and baboons^21^. In addition, common marmosets are also characterized by their small body size, low zoonotic risk, ease of handling and maintenance, multiple litters, and lower costs^21^,^22^. Thus, the use of marmoset to model the effect of aging on MaSC functions would be advantageous over both the conventional rodent models such as mouse or rat as well as other long-lived non-human primates such as rhesus macaque.

Currently, little is known about mammary stem/progenitors in any non-human primate species. The goal of the present study was to characterize mammary stem/progenitor cells in the common marmosets, and determine whether the stem/progenitor cells change with age. We adopted a suite of methods that have been used to characterize stem/progenitors in mouse and human mammary glands, and successfully identified four types of primary stem/progenitors cells residing in the marmoset mammary glands. Similar observations were also found in another non-human primate, the olive baboon (*Papio hamadryas Anubis*). Importantly, we found that luminal-restricted progenitors in common marmoset decreased significantly with age, while myoepithelial-restricted or bipotent progenitors remained constant with aging.

## Results

### Basic characteristics of marmoset mammary gland

**Figure 1:**
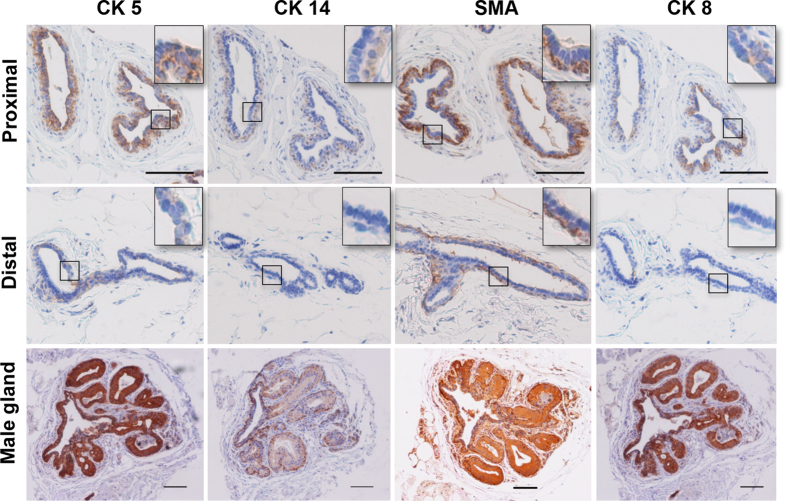
Mammary gland sections from female (proximal and distal to nipple) and male common marmosets stained for K5, K14, SMA, and K8. Scale bars, 100 μm.

Female marmosets have two symmetrically located flat thoracic mammary glands. The cauliflower-like ductal system of adult gland is composed of several main ducts, which branch into many smaller ducts and end with lobules (Fig. S1). We first examined different basal and luminal lineage markers for ducts promixal (main ducts) and distal (secondary or tertiary ducts) to nipple. For basal markers, we used cytokeratins K5, K14 and smooth muscle actin (SMA). K5 was reported as a partner of K14 in keratin filaments, and only stains basal cells in mouse mammary gland^23^, but stains both basal and luminal cells in human breast tissue^24^,^25^. For the marmoset mammary ducts, K5 stained strongly in main ducts close to nipple and mainly in basal cells, but also stained positive for some luminal cells (Fig. 1). However, for peripheral ducts distal to nipple, K5 positive cells were mainly confined to basal layer (Fig. 1). K14 was stained positive exclusively for basal cells in the main ducts near nipple, but stained negative for all cells in the secondary or tertiary ducts that are distal to nipple (Fig. 1). SMA, reported to be a basal marker for differentiated myoepithelial cells, stained positive exclusively in all basal cells of all ducts in the present study (Fig. 1). Cytokeratins K8, K18, mucin 1 (Muc1), and epithelial cell adhesion molecule (EpCAM) were used as luminal markers. K8 and K18 were reported as a paired keratin filaments and were restricted to luminal cells both in mouse^23^ and human mammary glands^25^,^26^. In marmosets, K8 stained mainly in luminal cells and a few basal cells but only in main ducts near nipple area, no positive stained cells were found in peripheral ducts (Fig. 1). K18 staining pattern was similar to that of K8, and Muc1 was stained negative (data not shown). Whereas in adult male marmoset mammary gland, which only has rudimental main ducts surrounding the nipple, all basal and luminal cells were positive for K5, K14, SMA and K8. For K14, basal cells were stained much stronger than luminal cells (Fig. 1).

### Primary epithelial cells can be fractionated into distinct cell populations using integrin α6

**Figure 2:**
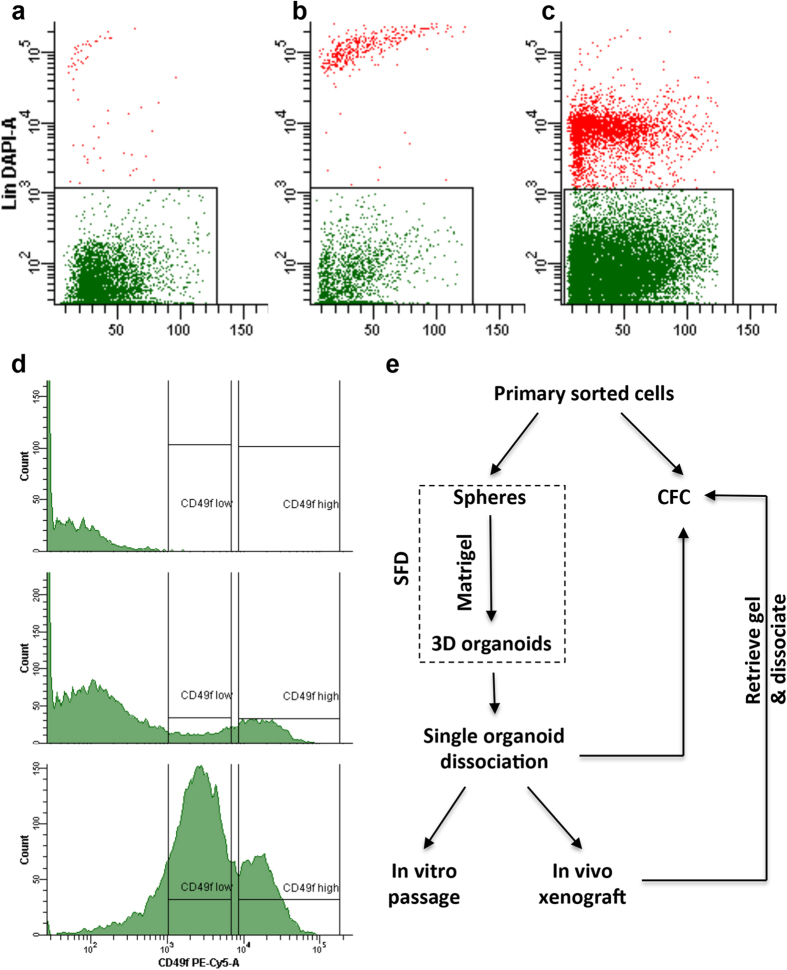
Representative FACS plots showing different profiles of endothelial (CD31) and hematopoietic (CD45 and CD235a) lineage positive cells (shown as red dots in Panel a–c) and gating strategy based on different expression levels of CD49f in the Lin^−^ mammary epithelial cells (Panel d). (**e**) A schematic diagram shows experimental flow for sorted cells. SFD refers to sphere formation and differentiation assay, and CFC refers to colony formation on irradiated NIH3T3 cells.

For human and mouse stem/progenitor cell isolation and enrichment, mammary glands were first digested with a combination of collagenase/hyaluronidase into a single cell suspension. Epithelial cells were then enriched by gating on endothelial (CD31) and hematopoietic (CD45 and CD235a) negative cells (also termed as lineage-depleted or Lin^−^ cells) with flow cytometry. Basal and luminal stem/progenitor cells were further separated based on a combination of two commonly used surface marker expression levels of CD24 (mouse) or EpCAM (human) and integrin α6 (CD49f)^27^,^28^. We applied this exact method for stem/progenitor cell isolation in marmoset mammary glands. Interestingly, we found very few Lin positive cells (Fig. 2a,b) in most samples, but one sample exhibited high Lin positive cell population (Fig. 2c). This finding is consistent with limited blood vessels in H&E stained tissue sections (data not shown). For separation of luminal (CD24^+^/EpCAM^+^CD49f^lo^) vs. basal cells (CD24^lo^/EpCAM^lo^CD49f^hi^), we did not observe positive cell population for either CD24 or EpCAM, which is likely due to the lack of antigenicity of the marmoset CD24 and EpCAM to the antibodies we used. This is consistent with the negative staining of EpCAM (data not shown) in marmoset mammary ducts. We also tested these antibodies with epithelial cells isolated from olive baboon mammary glands, and found similar observations as for the marmosets (data not shown). As a result, CD49f was used for cell profiling. The expression level of CD49f separates Lin^−^ cell into three distinct fractions: CD49f negative, CD49f low, and CD49f high cells (Fig. 2d). Among 10 marmosets examined, we found glands from eight females were dominated by a large population of CD49f negative cells (~75%) with smaller populations of CD49f high cells (~14%) and CD49f low cells (~9%) (middle panel in Fig. 2d). However, the glands from two females were characterized by a large fraction of CD49f low cell fraction (lower panel in Fig. 2d).

### CD49f positive cells contain *in vitro* colony forming cells

**Figure 3:**
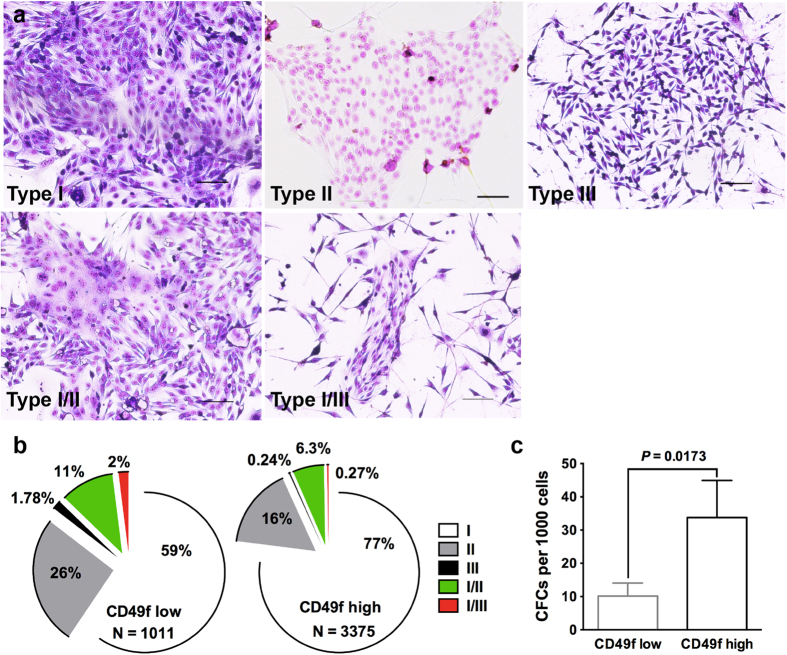
(**a**) Representative images showing three types of distinct *in vitro* colonies and two types of mixed colonies formed by FACS-sorted CD49f low and CD49f high cells. Scale bars, 100 μm. (**b**) The distribution of different types of colonies in CD49f low vs. CD49f high cells. The total colony number (N) is the sum of colonies formed by 100,000 cells from 10 animals (10,000 cells/animal). (**c**) The efficiency of colony forming cells (CFCs) in CD49f low and CD49f high population (n = 10, mean ± SE).

To assess functional difference of these distinct cell populations, we adopted a series of *in vitro* and *in vivo* assays used previously for mouse or human stem/progenitor cells (Fig. 2e). In particular, the colony forming cell (CFC) assay provides an *in vitro* readout for progenitor cells that can form discrete colonies^29^,^30^. In the present study, isolated Lin positive and CD49f negative cells barely formed any *in vitro* colonies when these cells were plated on irradiated NIH3T3 coated wells (data not shown). For the sorted CD49f low and high cells, we observed three types of morphologically distinct colonies and two types of mixed colonies (Fig. 3a; Table 1). Type I colonies are characterized by a compact arrangement of the cells with large variation in colony size (ranged from >50 cells to 1000s), and type II colonies are characterized by a less closely arranged cells and fewer cells in colony size (ranged from >50 to 100s cells), but both types of colonies have indistinct cell borders and a smooth outer colony boundary. Type III colonies are characterized by teardrop-shaped cells without a clear colony boundary (Fig. 3a). The morphological appearance of type I and II colonies resembles the luminal-restricted colonies found in human epithelial cells, and the type III colonies resemble the myoepithelial-restricted colonies in humans^30^,^31^. Immunocytochemistry staining of these colonies with various basal and luminal markers revealed limited differences among the three types of colonies with the exception that K8 and K14 are more uniformly expression in the cells of the type I and II colonies than in the cells of the type III colonies (Figs S2 and S3). The mixed colonies were composed mainly of type I and II or type I and III mixtures (Fig. 3a). The distribution of different types of colonies varied among individual animals (Fig. S4). When combined all colonies formed by 10,000 cells/animal from all 10 marmosets, type I colony was the most dominant one accounting for 59% of all types of colony in CD49f low cells (n = 1011 colonies) and 77% in CD49f high cells (n = 3375 colonies) followed by type II and type I/II mixed colonies (Fig. 3b). Type III and type I/III mixed colonies are very rare, together only accounted for <4% of total CFCs and also seemed more prevalent in CD49f low cells. Another interesting observation is that CD49f high cells have significantly higher colony formation efficiency averaging around 34 CFCs per 1,000 cells (with a range between 6 and 123, n = 10 animals) when compared with an average of 10 CFCs per 1,000 cells (with a range between 1 and 40, n = 10 animals) in CD49f low cells (Fig. 3c).

### CD49f positive cells contain sphere formation and differentiation initiating cells

**Figure 4:**
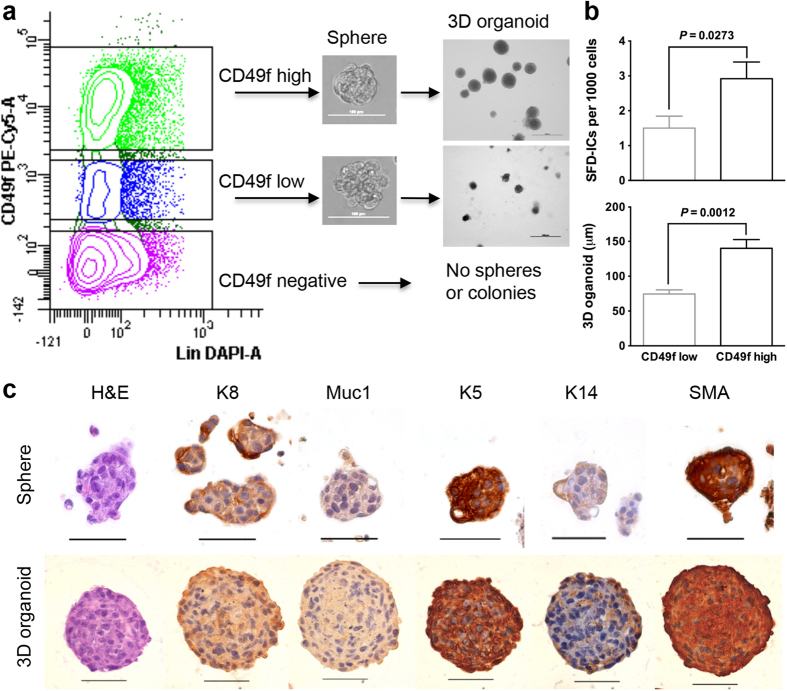
(**a**) Scheme of the sphere formation and differentiation (SFD) assay showing spheres and 3D organoids formed by sorted CD49f low and high cells, but not CD49f negative cells. (**b**) The efficiency of SFD initiating cells (SFD-ICs) (n = 10, mean ± SE) and size of 3D organoids (with a range between 16 and 242 organoids per animal, n = 7 animals) derived from FACS sorted CD49f low and high cells. (**c**) Spheres and 3D organoids derived from CD49f high cells stained for K8, Muc1, K5, K14, and SMA. Scale bars, 50 μm.

In rodents, the sphere formation and differentiation (SFD) assay was used for the identification of mouse mammary stem/progenitor cells *in vitro* based on their ability to form mammospheres with subsequent proliferation and differentiation in Matrigel culture^32^. We also adopted this method for the marmoset mammary cells. Similar to the results from the CFC assay, we obtained very few spheres from Lin positive and CD49f negative cells and none of them can form 3D orgnanoid in subsequent Matrigel culture. However, about 43% of spheres derived from CD49f low and 97% of spheres derived from CD49f high cells formed 3D organoids with solid appearance (Fig. 4a). Similar to colony formation efficiency in the CFC assay, the frequency for SFD initiating cells (SFD-ICs), which are the cells that can form spheres and proliferate and differentiate into 3D organoids, was also significantly higher in CD49f high cells (2.9 ± 0.5, n = 10 animals) than that in CD49f low cells (1.5 ± 0.3) (Fig. 4b). Although we did not observe any difference in sphere size between spheres derived from CD49f low vs. high cells, the resultant 3D organoids from spheres formed by CD49f low cells (74 ± 6 μm, n = 7 animals) were significantly smaller than those from CD49f high cells (140 ± 12 μm) (Fig. 4b). Despite this, we did not observe any significant difference in basal and luminal marker staining for spheres and 3D organoids between CD49f low vs. high cells. All cells in spheres and 3D organoids were positive for K8, K5, and SMA (Fig. 4c). All cells in spheres were negative for Muc1, but with partial stain for K14 (Fig. 4c). For cells in 3D organoids, the outer layers were positive for K14, and the center were mixed with K14 positive and negative cells (Fig. 4c). The staining patterns for these markers in 3D organoids resemble that of *in situ* staining of mammary ducts proximal to nipple shown in Fig. 1.

### SFD-ICs demonstrate self-renewal and multi-lineage differentiation *in vitro* and *in vivo*

**Figure 5:**
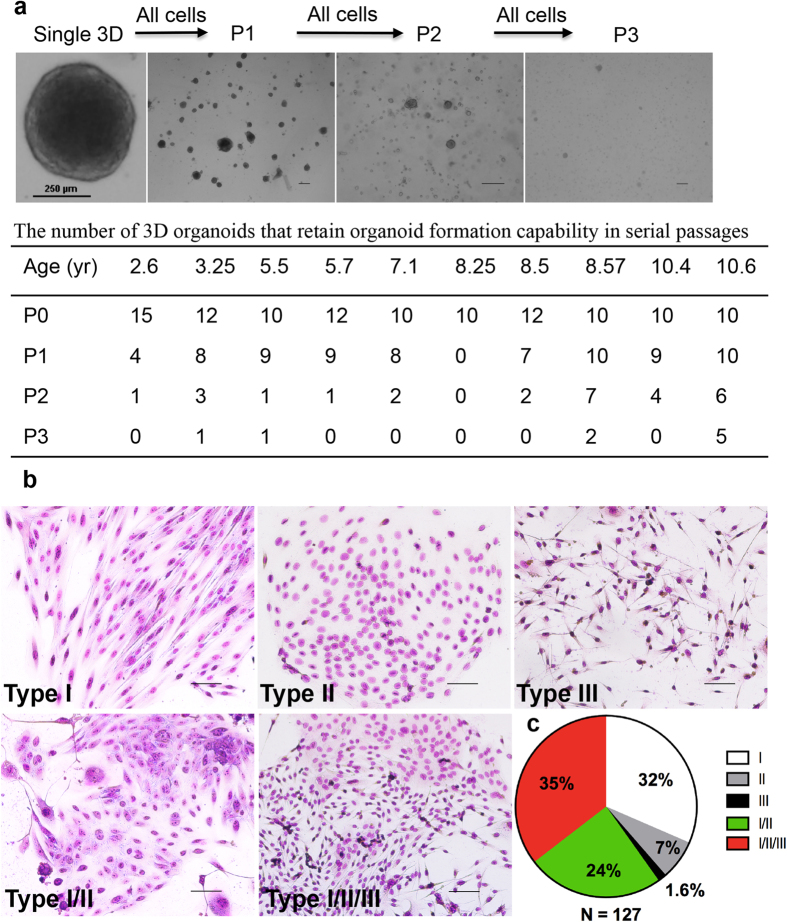
(**a**) *In vitro* serial passage of 3D organoids reveals that most primary 3D organoids (P0) can be passaged to P1 and some of them can be passaged to P2 or P3. Scale bars, 250 μm. (**b**) Representative images showing distinct colonies formed by cells dissociated from individual primary 3D organoid. Scale bars, 100 μm. (**c**) The pie chart shows the distribution of different types of colonies formed by cells dissociated from a total of 127 primary 3D organoids from 8 marmosets.

In the mouse study, SFD-ICs from basal cells have been shown to contain MaSCs that could efficiently regenerate a mammary tree upon *in vivo* transplant of single 3D organoid into cleared fat pads in recipient mice, and the regenerated mammary glands were able to produce milk upon pregnancy and capable of self-renewal generating new glands upon serial transplant^32^. To test whether the 3D organoids generated in the present study with marmoset epithelial cells also contain stem/progenitor cells, we first tested their self-renewal and multi-lineage differentiation *in vitro* (Fig. 5a). Serial passage of cells dissociated from single 3D organoid revealed that majority of them, except for those from the youngest marmoset, could at least be passaged for one generation, and few of them could also be passaged for three generations (Fig. 5a), indicating that these 3D organoids are capable of self-renewal though with rather limited potential. We also plated cells dissociated from these primary 3D organoid for the CFC assay to check multi-lineage differentiation. Individual 3D organoids derived from CD49f high cells were randomly selected (10–20 organoids per animal), dissociated and plated separately for the CFC assay. These 3D organoid-derived cells generated *in vitro* colonies morphologically similar to those from primary CD49f positive cells (Fig. 5b). The IHC staining for basal markers of K5, K14, and SMA was also same except for CD10, which was positive for colonies from primary sorted cells, but was negative for colonies derived from cells dissociated from 3D organoids (Figs S5 and S6). Interestingly, the myoepithelial-restricted colonies formed this time were mainly negative for K8, which is different from the same colonies formed by primary sorted cells (Fig. S6 vs. Fig. S3). Except for a few organoids, cells dissociated from most organoids formed either single or mixed colonies in the CFC assay. In a total of 127 organoids dissociated (10–20 organoids per animal from 8 animals), roughly 32% formed exclusively type I colonies, 7% formed exclusively type II colonies, 1.6% formed exclusively type III colonies, 24% formed colonies contain type I and type II, and 35% formed colonies contain all three types (Fig. 5c). These data suggest that 3D organoids contain different progenitors that can give rise to exclusive luminal or myoepithelial lineage cells as well as cells of both lineages.

**Figure 6:**
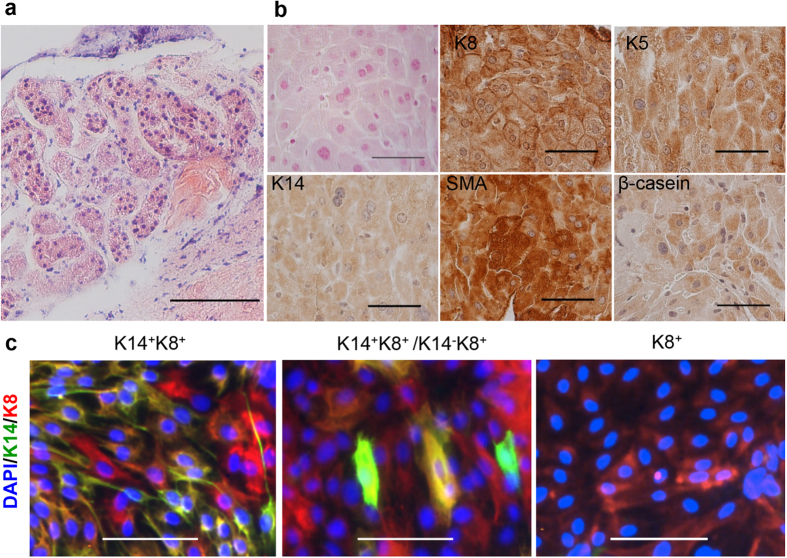
(**a**) Lobular-like structures formed in the collagen gels from renal capsule xenograft in the NOD/SCID mice. Scale bars, 100 μm. (**b**) Collagen gels harvested from pregnant recipient NOD/SCID mice stained for K8, K5, K14, SMA, and β-casein. Scale bars, 50 μm. (**c**) *In vitro* colonies formed by cells dissociated from the collagen gels and stained for K14 and K8. Left panel shows colonies mainly contain K14^+^K8^+^ double positive cells; middle panel shows colonies contain mixture of K14^−^K8^+^ and K14^+^K8^+^ cells; right panel shows colonies only contain K8^+^ cells. None of these colonies were positive for ESA (data not shown). Scale bars, 100 μm.

To test MaSC function *in vivo*, the cleared fat pad transplantation assay was generally used for mouse MaSCs, and for human MaSCs, researchers either use humanized mouse fat pads by colonizing cleared fat pads of immunodeficient mice with human fibroblasts^33^ or a renal capsule xenotransplantation assay^34^ due to the fact that human mammary epithelial cells do not readily proliferate in the adipose environment of the mouse mammary fat pad. In the present study, because marmoset epithelial cells behavior more like human epithelial cells in terms of *in vitro* sphere and colony formation, we thus adopted the renal capsule xenotransplantation assay for *in vivo* MaSC function analysis. This assay also circumvents the need to modify the mouse mammary fat pad and is rapid and economical to perform^34^. Specifically, we implanted cells dissociated from single 3D organoid together with irradiated mouse embryonic fibroblasts in collagen gel plugs into the renal capsule of hormone-supplemented immune-deficient female NOD/SCID mice. The gels harvested 4 weeks later after transplant showed lobule-like structures (Fig. 6a) that seem to deviate from normal mammary ducts, which may have resulted from a suboptimal incubation time adopted straight from the human protocol^34^. Despite this, cells of these structures were stained positive for K8, K5, K14, and SMA in virgin recipient and positive for β-casein in pregnant recipients (Fig. 6b), indicating *in vivo* self-renewal and multi-lineage differentiation. We also further dissociated structures contained in the collagen gels into single cell suspension and plated these cells for CFC assay as previously used for human stem/progenitor cell identification^34^,^35^. The *in vitro* colonies formed contained K8^−^K14^−^, K8^+^K14^−^, and K8^+^K14^+^ cells (Fig. 6c). Cells dissociated from different gels yielded a wide range of CFCs with some gels resulted no colonies (Fig. S7), yet the colony formation efficiency of cells dissociated from these *in vivo* xenograft gels are comparable to those reported in human MaSC studies^34^,^35^. Together, our *in vitro* and *in vivo* results indicate that SFD-ICs derived from marmoset CD49f positive cells contain stem/progenitor cells that are capable of self-renewal and multi-lineage differentiation.

### SFD-ICs exhibit no change during aging yet CFCs decreases with age

**Figure 7:**
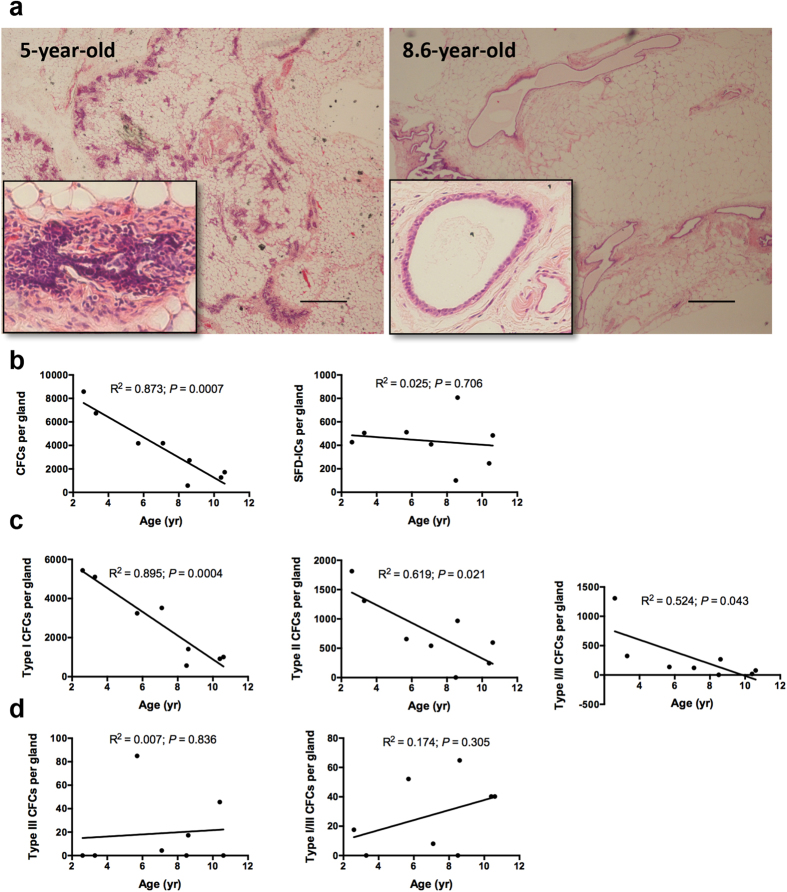
(**a**) Mammary gland H&E sections showing dense ductal structures in a 5-year old female marmoset and scarce ductal structures in a 8.6-year old female marmoset (inserts are high magnification of ductal structures; scale bars, 500 μm). (**b**) Linear regression analysis between the total number of colony forming cells (CFCs) and SFD initiating cells (SFD-ICs) per gland and animal age, and between the total CFCs per gland and animal age for type I, type II, and type I/II mixed colonies (shown in panel c) as well as for the type III and type I/III mixed colonies (shown in panel d).

It is known that stem cell function may decline with age, and indeed our own studies with mouse stem/progenitor showed decreased self-renewal and multi-lineage differentiation and increased transformation potential with age (our unpublished data). The marmosets used in this study ranged from 2.6 to 10.6 years old, an equivalent of age span from 20s to later 60s in humans^21^,^36^. H&E tissue sections showed more abundant duct and lobule structures surrounded by dense fibrous connective tissues in relatively young animals around 5 yr old (2 animals examined) when compared with animals >8 yr old (5 animals examined) where less lobule structures, loose fibrous connective tissue, and more abundant fatty tissues were seen (Fig. 7a). These morphological changes have also been observed in aged human breasts^37^.

Though mammary tissues in this study were collected from necropsy animals, most animals were culled for colony management purpose or sacrificed as control animals for a separate project, and were relatively health at the time of sacrifice (Table 2). In addition, these females were housed at the same conditions and had similar ovarian cyclical activity, and majority of them are non-parous (Table 2). We thus used all of them except for the two animals that were culled for health reasons to gauge the effect of aging on stem/progenitor cell. We calculated the total number of CFCs and SFD-ICs per gland for each animal based on CFC or SFD forming efficiency and total number of sorted cells for each cell fraction (see formula [1]–[3] in methods). Our analyses revealed a linear decrease of CFCs with aging, yet no age effect on SFD-ICs (Fig. 7b). We further looked into the relationship between the number of different types of CFCs and age, the linear decrease associated with aging was mainly contributed by luminal-restricted type I and II colonies (Fig. 7c). For myoepithelial-restricted type III colony and the type I and III mixed colonies, we actually saw a trend of increase with age, though it was not statistically significant (Fig. 7d). These findings suggest age related decrease of luminal-restricted progenitors.

## Discussion

In the present study, by adopting the methods used for stem/progenitor cell identification in mouse^28^,^32^ and human mammary tissues^24^,^27^,^34^, we characterized for the first time the mammary stem/progenitor cells in the common marmoset, a non-human primate. Similar to findings in mice and humans, stem/progenitor cells in marmosets were highly enriched in endothelial (CD31) and hematopoietic (CD45 and CD235a) lineage-depleted (Lin^−^) CD49f positive cells, in particular the CD49f high cells. The sorted CD49f positive cells were able to form morphologically distinct epithelial cell colonies when cultured on plates pre-seeded with irradiated NIH3T3 fibroblasts, and also were capable of forming mammospheres in suspension culture and subsequent sphere proliferation/differentiation into 3D organoids in Matrigel culture. Most importantly, these 3D organoids were found to contain stem/progenitor cells that can undergo self-renewal and multi-lineage differentiation both *in vitro* and *in vivo*. These findings demonstrate that the mammary stem/progenitor cells in common marmoset can be isolated and quantified with established *in vitro* and *in vivo* assays used for mouse and human studies.

Mammary stem cells are usually quantified using the limiting dilution *in vivo* transplantation assay, however, recently published lineage tracing data indicated a more restricted unipotent cell fate for MaSC tested *in situ* than those tested in transplantation systems^38^,^39^,^40^. These findings raise significant concern about using *in vivo* transplant assay as the gold standard to define stem cell property^41^. Although the most recent findings demonstrated the presence of multipotent MaSCs, it is generally agreeable that mammary epithelia are maintained by unipotent basal and luminal MaSCs^42^,^43^. Yet, current *in vivo* transplantation system was only able to assay basal MaSCs because luminal MaSCs (or progenitors) cannot repopulate the mammary gland in the cleared fat pad as efficiently as that of basal MaSCs^28^,^44^, which leaves *in vitro* colony forming assay the only option for luminal progenitor identification and quantification^41^. Given all these concerns, we thus mainly rely on these *in vitro* CFC and SFD assays for stem cell identification, which provide additional advantages over the *in vivo* transplantation assay as they not only allow the assessment of both basal and luminal stem/progenitors, but also are cost-effective due to no further requirement of using excessive recipient animals.

Based on the distinct colonies formed in the CFC assay, we deduced that there are at least four types of stem/progenitors residing in the marmoset mammary glands: two luminal-restricted progenitors (type I and II), one myoepithelial-restricted progenitor (type III), and one bipotent progenitor (type I and III mix) that give rise to colonies with luminal cells at the center and myoepithelial cells at the periphery. We are uncertain about an additional progenitor that may contribute to the mixed colonies contain both type I and type II as these mixed colonies could resulted from two luminal-restricted progenitors that were very close to each other and proliferated into each other. On the other hand, for the mixed colonies with the luminal cells at the center and myoepithelial cells at the outer layer, studies with human cells have proved that these colonies originated from single cells^30^,^45^. Our observation of four stem/progenitor cells residing in the mammary glands may be generalized to all species as all mammary glands have the same function. Indeed, our preliminary findings with another non-human primate, the olive baboon, revealed exactly the same four types of *in vitro* colonies in the CFC assay for cells isolated and sorted in a similar way as those from the marmosets (Fig. S8). These same types of morphological distinct colonies have been reported in mammary epithelial cells isolated from human breast tissue initially by Dr. Chang’s group^31^,^46^,^47^, and later have also been confirmed by others^30^. The two luminal-restricted progenitors may correspond to the ER^+^ and ER^−^ progenitor identified recently^44^, the myoepithelial-restricted progenitor may correspond to the unipotent basal MaSCs^40^, and the bipotent progenitor could be the multi-potent MaSCs identified recently^42^,^43^. Future studies are necessary to pinpoint the exact *in vivo* counterparts for these *in vitro* colonies to allow precise constructing of the stem cell hierarchy within the mammary gland^41^.

Though both the CFC and SFD assays allow us to identify stem/progenitor cells *in vitro*, these two assays yielded different frequency for different progenitors. In the present study, the CFCs contained in sorted primary CD49f positive cells are predominantly luminal-restricted progenitors with the myoepithelial or bipotent progenitors only accounting for less than 4% in all formed colonies. However, when the 3D organoids derived from SFD-ICs were dissociated and plated for colony formation, approximately 37% of these SFD-ICs are myoepithelial or bipotent origin. Similar findings were also found in baboons (data not shown). Together, these results indicate that for the sorted primary cells, the CFC assay maybe biased to preferentially detect luminal-restricted progenitors and the SFD assay maybe biased to preferentially detect bipotent progenitors. Previous findings with mouse mammary tissue also support this speculation. For example, in the young C57BL/6J mice, the CFC assay produced very few colonies from sorted basal cells (<1 colony per 5000 cells), but yielded abundant colonies from sorted luminal cells (100–300 colonies per 1000 cells). While for the same C57BL/6J mice, the SFD assay produced around 7 spheres per 1000 basal cells, and 47 spheres per 1000 luminal cells, a much smaller difference in colony/sphere formation efficiency between basal and luminal cells in the SFD assay than that of CFC assay^32^,^48^. Thus, these two *in vitro* assays may provide disproportional readouts for different stem/progenitor cells, and it is unknown which assay is more faithful in reporting true stem/progenitor cells *in vivo*. More recently, similar findings have also been reported in human mammary glands where growth of epithelial cells as mammospheres enriched for both alveolar and ductal progenitor activity, and growth in suspension as floating colonies over adherent plates preferentially enriched for alveolar progenitor activity^49^. Clearly, future studies are necessary to further delineate the relationship between *in vitro* readouts of various stem/progenitor assays and their *in vivo* counterparts.

In the mouse study, the 3D organoids formed by spheres exhibited distinct morphological difference between luminal vs. basal stem/progenitors with the former forming hollow-like structures and the latter forming solid structures^32^. However, the 3D organoids derived from the marmoset or baboon mammospheres all formed solid structures, and no hollow structures were observed. This phenomenon is similar to human stem/progenitor cells where both luminal and basal cells formed round 3D organoids between 50 and 100 μm^27^. However, in the human case the solid 3D organoids will become hollow when supplied with prolactin^24^. We did not observe this in the present study when 3D organoids were supplied with the same concentration of prolactin (data not shown). Nevertheless, the dissociated cells from these 3D organoids formed distinct epithelial colonies when they were subjected to the CFC assay, suggesting that these 3D organoids were initiated by different stem/progenitors.

If we assume that the CFC assay can better estimate luminal-restricted progenitors and the SFD assay can better estimate the myoepithelial-restricted and bipotent progenitors, our present study showed that in marmosets luminal-restricted progenitors decreased significantly with aging, yet myoepithelial-restricted or bipotent progenitors remained constant or increased moderately with aging. This result seems consistent with our observation of the pronounced paucity of lobules in the old marmoset glands. In our mouse study, we observed similar decrease of *in vitro* colonies formed by luminal cells, yet a significant increase of SFD-ICs from basal cells from old (25 months) mammary glands when compared to young (4–6 months) mammary glands, where gene enrichment analysis and immunofluorescence staining indicated a potential mechanism of luminal cells undergo luminal-to-basal phenotypic changes during aging (unpublished results). It is currently unknown what the pathological implication is associated with these changes of basal and/or luminal progenitors in aged glands. However, when compared with differentiated cells, stem/progenitor cells are believed to be more likely to become the cancer initiating cells given their relatively long-lived, self-renewal and multi-lineage differentiation properties, which are shared by cancer initiating cells. Thus, the age-associated changes of mammary stem/progenitor cells may be the underlying etiology of age-associated mammary tumorigenesis. However, the causes for these age-associated changes are unknown. Aging associated pathogenesis is a complicated process characterized by many different molecular mechanisms^3^. In our mouse study, we observed activated inflammatory signals, immune responses and elevated p16 expression in aged stem/niche cells (unpublished results), which may implicate a possible role of chronic inflammation in aging-induced changes of mammary stem/progenitor cells. Future studies are necessary to confirm this hypothesis in mice, marmosets and humans.

Integrin α6 (CD49f), although no functional role in mammary gland development^50^, has been routinely used for effective separation between basal and luminal cell in the mouse and human mammary cells^27^,^28^. In the present study, we were able to separate Lin negative marmoset mammary cells into three distinct fractions based on CD49f expression level, and most CFCs or SFD-ICs were enriched in CD49f high cells. A distinct separation between luminal and basal cells, similar to what have been observed in mice or humans, was not achieved in marmosets. In baboons, Lin negative mammary cells can be fractionated into CD49f negative and CD49f positive cells, and the CD49f negative cells appeared to be dominated by luminal-restricted progenitors while the CD49f positive cells were mostly myoepithelial-restricted progenitors (Fig. S8). The surface markers of CD24 and EpCAM are generally used in combination with CD49f for luminal and basal cell separation^27^,^28^; however, both markers did not work in either our marmoset or baboon tissues despite their high homology to their human counterparts at the protein level (>75% for CD24 and >90% for EpCAM). Since we could not obtain positive staining for CD24 and EpCAM of marmosets or baboons after testing their antibodies from various vendors, it is possible that the mouse or human antibodies we tested were unable to recognize marmoset and baboon antigens. Future studies can explore the utilities of other mammary stem/progenitor markers such as stem cells antigen 1 (Sca 1) and promin-1 (CD133) in non-human primates. The limited availability of antibodies specific for non-human primate antigens is a great challenge for using these models for mammary stem cell research. Similarly, for the various types of *in vitro* colonies, it was hard to tell them apart based on traditional basal and luminal markers used for mouse or human mammary cells as all marmoset mammary epithelial cells stained positive for K5, K14, SMA, and K8. In baboons, cells in luminal-restricted colonies were characterized by K8^lo^K14^+^ (C1 colony) or K8^+^K14^−^(C2 colony), cells in myoepithelial-restricted colonies were K8^+^K14^+^ (C3 colony), and cells in mixed colonies were K8^+^K14^lo^ in the middle and K8^lo^K14^hi^ in the peripheral (Fig. S9). In humans, cells in luminal-restricted colonies only express Muc1, K8/18, EpCAM and K19, and do not express K14, while cells in myoepithelial-restricted colonies express K14, but not MUC1, EpCAM and K19, and cells in mixed colonies typical express luminal markers in the center and basal markers in the periphery with some cells express both K14 and K18^30^. Non-human primate specific antibodies may allow more clear molecular characterization of these morphologically distinct *in vitro* colonies formed by the marmoset or baboon mammary stem/progenitor cells. Alternatively, it is possible that current *in vitro* culture conditions optimized for human mammary cells may inhibit or do not allow proper lineage marker expression for mammary cells from the marmosets or baboons^17^.

It is worthy noting that the lack of antigenicity of CD24 and EpCAM will not jeopardize our findings based on *in vitro* assays performed on CD49f low vs. high cells in marmosets as most of these assays were developed for stem/progenitor cell characterization in unsorted primary epithelial cells^24^,^45^. For human mammary epithelial cells, because both Lin^+^ (CD31^+^CD45^+^ CD235a^+^) and stromal (Lin^−^EpCAM^−^CD49f^−^) cells do not form colonies in the CFC or SFD assays (our own observations), and luminal and basal stem/progenitors give rise to distinct colonies *in vitro*, thus pre-separation of luminal and basal cells via FACS sorting is not necessary for *in vitro* colony forming assays, which is different from mouse epithelial cells where stromal cells can form spheres via cell-cell aggregation, and the use of defined luminal and basal cell populations is a prerequisite for accurate enumeration of stem/progenitor cells^32^. We did not observe any contributions of *in vitro* colonies from Lin^+^ or stromal cells in common marmosets, suggesting epithelial cells in non-human primates behavior more like humans than rodents.

In summary, despite some limitations, our study show that mammary stem/progenitor cells in non-human primates such as the common marmoset and baboon can be characterized by the current *in vitro* and *in vivo* methods used in mouse and human MaSC studies, and future studies employing these animal models for mammary stem/progenitor cell and breast cancer research are therefore feasible.

## Materials and Methods

### Animals

Marmosets and baboons were housed at one of the National Primate Research Center and all mammary tissues were obtained from necropsy animals. A total of 10 marmosets were used in this study and the majority of the animals was sacrificed for management purposes or as control animals for a separate study and was judged to be in good health at the time of sacrifice (Table 2). Mammary tissues (two glands per animal) were collected and transported from the primate center to the lab on ice in PBS. Animal care and use were conducted according to established guidelines approved by the Institutional Animal Care and Use Committee at the Southwest National Primate Research Center, and all experimental protocols were approved by the same committee.

### Mammary cell preparation

Mammary tissue was washed 5 times with PBS and then minced into small pieces with a scalpel in a glass petri dish. Minced tissue were transferred into a 50-mL tube containing dissociation medium (1 part 10x collagenase/hyaluronidase [Catalog No. 07912] and 9 parts EpiCult-B complete medium [Catalog No. 05620] supplemented with 5% fetal bovine serum [FBS] and 0.05 mg/mL gentamicin from StemCell Technologies, Vancouver, Canada) for 16h at 37 °C in a 5% CO_2_ incubator. The digested glands were then processed to single cell as previously described^32^. In brief, the resultant organoid pellet was processed sequentially in 0.64% NH_4_Cl, 0.25% trypsin-EDTA, and 5 mg/mL dispase with 0.1 mg/mL DNase I. Cell suspension was filtered through a 40-micron mesh before used for antibody staining.

### Antibodies

Antibodies for flow cytometric analysis included anti-CD24-FITC (Biolegend), anti-EpCAM-FITC (Stemcell Technologies), anti-CD49f-PE/Cy5 (BD bioscience), and biotinylated anti-CD31/CD45/CD235a (Biolegend). Pacific Blue-conjugated streptavidin (Invitrogen) was used to visualize the biotinylated antibody cocktail. Antibodies used for IHC/ICC include CK5 (Abcam 1:1000), CK8 (Abcam 1:500), CK14 (Abcam 1:200), CK18 (Abcam 1:200), CD10 (Abcam 1:50), SMA (Abcam 1:200), and ESA (Leica 1:200).

### Cell labeling and flow cytometry sorting

We adopted the human MaSC isolation protocol^27^ in the present study. Cells were enriched and isolated from endothelial (CD31) and hematopoietic (CD45 and CD235a) lineage-depleted (Lin^−^) mammary epithelial cells using cell surface markers of EpCAM and integrin α6 (CD49f). In details, cells were first incubated with biotinylated CD31/CD45/CD235a antibody cocktail on ice for 15 min. After washing with HBSS supplemented with 2% FBS, cells were incubated with anti-EpCAM-PE, anti-CD49f-PE/Cy5, and streptavidin-PB on ice for 10 min. After one more washing, cells were sorted (FACSAria IIIu, BD Bioscience) according to the gate illustrated in Fig. 2a,b.

### Colony forming cell (CFC) assay

The CFC assay was used to identify progenitor cells that can form *in vitro* colonies within the mammary cells in both mouse and human mammary glands^34^. In the present study, we plated 5,000 mammary cells into each well of the 6-well plates containing MMS medium (see below for details) supplemented with 5% FBS in the presence of ~8 × 10^4^ irradiated NIH-3T3 cells. Two wells (a sampling of 10,000 cells) were plated for each cell fraction per animal. After 24 h the media was replaced with serum-free MMS medium, and 8 days later the colonies were fixed with 100% cold methanol for 1 min, stained with 10% Giemsa for 30 min, and counted.

### Sphere formation and differentiation (SFD) assay

In the present study we also adopted the SFD assay developed for mouse MaSC identification^32^ for stem/progenitor cell characterization in marmosets and baboons. The detailed method has been described previously^32^. In brief, sorted cells were cultured in ultralow attachment 96-well plates (Corning) with human MammoCult complete medium (StemCell Technologies) supplemented with 2% B27 without vitamin A (Invitrogen), 20 ng/mL bFGF, 20 ng/mL EGF, 10 μg/mL heparin, 10 μg/mL insulin, 1 μg/mL hydrocortisone, 50 μg/mL gentamycin (referred to as mammosphere or MMS medium) at 37 °C in a 5% CO2 incubator. Cells were plated at two densities of 10,000 and 20,000 cells/well. After 7–10 days of suspension culture, spheres were counted and about 50 spheres were handpicked under a dissecting microscope and resuspended in 60 μl chilled Matrigel (BD Biosciences) for sphere differentiation. The sphere-Matrigel drop was allowed to solidify inside a 37 °C incubator for 15 min, covered with MMS medium supplemented with 5% FBS, and incubated at 37 °C for 9 days before examination.

### Stem/progenitor cell quantification

In this study, we refer to cells capable of forming *in vitro* colonies operationally as colony forming cells (CFCs), and cells that are capable of forming spheres in suspension culture and subsequently differentiating into 3D organoids in Matrigel culture operationally as sphere formation/differentiation initiating cells (SFD-ICs). The CFCs or SFD-ICs per cell fraction was thus calculated as follows:

[1] Colony or sphere formation efficiency (CFE or SFE) = No. of colonies (spheres) per 1,000 cells.

[2] CFCs per cell fraction = CFE × total cell number in this fraction/1000.

[3] SFD-ICs per cell fraction = SFE × %sphere formed 3D organoids × total cell number in this fraction/1000.

The total CFCs or SFD-ICs per gland was the sum of CFCs or SFD-ICs in the CD49f low and high cell fractions.

### *In vitro* serial passage of 3D organoids

Primary 3D organoids (P0) were harvested at day 9 in culture by dissociating the Matrigel with 2.5 mg/mL dispase at 37 °C in a 5% CO_2_ incubator for 30 min. Dispase activity was stopped by adding sufficient (3x volume in excess) HBSS supplemented with 2% FBS, and individual 3D organoids were handpicked into 15-mL tubes (one organoid per tube). Single organoid was dissociated into single cell suspension by adding 500 μL 0.25% trypsin-EDTA and pipetting up and down for 3 min. The cell suspension was spun down and pellet resuspended in 10 μl HBSS supplemented with 2% FBS, mixed with 60 μl chilled Matrigel, and cultured by the sphere differentiation assay described above for 2 weeks (P1). The same process was repeated for continued 2^nd^ and 3^rd^ passages except that all organoids from P1 or P2 were used for subsequent passages.

### Whole mount staining

Whole mount staining of mammary glands was initially performed using standard carmine-alum procedures for mouse studies, however, the overall quality of these images were suboptimal due to low contrast between the mammary ducts and background. We then tried out other methods including the β-gal staining by using a senescence detection kit (BioVision), which generates reasonable whole mount images. Briefly, mammary glands were dissected from marmoset at the necropsy, spread onto glass slides, fixed in the fixative solution (provided by the kit) for 15 min, washed with PBST for three times, stained overnight in the staining solution, washed twice in PBST, washed twice in 100% methanol, and stored in 70% glycerol at 4 °C prior to imaging.

### Immunohistochemical staining

Fresh mammary tissues, spheres or 3D organoids (embedded in 1% agarose) were fixed in 10% neutral buffered formalin for 24~48 hr, dehydrated in ethanol and embedded in paraffin wax. Tissue sections of 4 μm thickness were deparaffinised and rehydrated through graded ethanol. Antigen retrieval was performed by heating in 10 mM sodium citrate (pH 6.0, 95 °C) for 10 min, followed by cooling on the bench. Endogenous peroxidase was inhibited by incubating sections with 3% H_2_O_2_ for 15 min and non-specific binding was blocked with 10% normal serum for 30 min at room temperature. The sections were incubated with primary antibodies overnight at 4 °C. Sections were then washed with 0.025% Triton X-100 in PBS (PBST) and incubated with biotin conjugated secondary antibodies for 1 hr at room temperature. After washing, sections were incubated with Sav-HRP for 30 min and stained with diaminobenzidine (DAB) for 15 min before dehydration and mounting.

### Immunocytochemistry staining

*In vitro* colonies formed on plastic plates were fixed in cold methanol for 5 min, washed with PBST, then blocked with 10% serum/1%BSA/0.3M glycine in 0.1% PBS-Tween 20 for 30 min. The colonies were incubated with primary antibodies diluted in 5% serum/1%BSA/0.3M glycine in 0.1% PBS-Tween 20 at 4 °C overnight, and then washed with PBST twice before incubated with secondary antibodies diluted in 1%BSA/0.3M glycine in 0.1% PBS-Tween 20 at room temperature for 1 hr. Colonies were then washed with PBST and stained with DAPI for fluorescent imaging.

### Preparation of collagen gels

The detailed method of collagen gel preparation can be found in previous publications^34^,^35^. In brief, collagen IV was neutralized by adding two parts (vol/vol) concentrated sodium hydroxide to 78 parts concentrated collagen solution and 20 parts 5× DMEM. The cell-gel mixture droplets were prepared by mixing 22 × 10^5^ irradiated (15 Gy) C3H 10T^1^/_2_ mouse embryonic fibroblasts with cells dissociated from single 3D organoid. The resultant cell suspension was pelleted and resuspended in 25 μl cold neutralized collagen. This cell-gel drop was then plated in a 35-mm petri dish, and incubated at 37 °C with 5% CO_2_ for 15 min before adding MMS medium. The gel drop is ready for transplantation.

### Renal capsule xenograft

The NOD/SCID nude mice were used as the recipient for renal capsule xenograft of marmoset mammary stem/progenitor cells. The detailed methods for this procedure can be found elsewhere^34^,^35^. In brief, we placed mice on anesthesia, removed the back hair, and swabbed the skin with 70% alcohol. A small incision (anterior to posterior) in the abdominal wall above one kidney was made and the kidney was exteriorized by applying gentle pressure on either side. We then lifted the kidney capsule from the parenchyma with fine forceps, made an incision of 2–4 mm wide, carefully inserted three gel drops under the capsule with a fire-polished glass pipette tip, sutured the incision on the abdominal wall, and repeated the same procedure on the contralateral kidney. A slow-release pellet containing 2 mg β-estradiol and 4 mg progesterone in MED-4011 silicone was inserted subcutaneously in a posterior position to produce sustained serum levels of these hormones. In a subset of recipient mice, mating was initiated 9 days after gel transplantation. Gel drops were retrieved 4 weeks post operation, directly fixed and paraffin embedded for antibody staining or dissociated into single cells for CFC assay as previously described^34^.

### Statistical analysis

Paired two-sided Student’s *t*-tests were used to compare differences between CFC, SFD-IC, and 3D organoid size between CD49f low vs. CD49f high cell population. Linear regression analysis was used to assess the effect of aging on CFCs and SFD-ICs. Results are presented as means ± SE and probability values of *P* < 0.05 were considered to be significant unless specified otherwise.

## Additional Information

**How to cite this article**: Wu, A. *et al*. Characterization of mammary epithelial stem/progenitor cells and their changes with aging in common marmosets. *Sci. Rep.* **6**, 32190; doi: 10.1038/srep32190 (2016).

## Supplementary Material

Supplementary Information

## Figures and Tables

**Table 1:** Summary of lineage marker expression in colonies formed by four different types of stem/progenitors.

	Stem/progenitor type			
Markers	Type I	Type II	Type III	Type I/III mix
K8	+	+	±	C+; P-low
K18	+	+	+	C+; P-low
Muc1	−	−	−	−
K5	+	+	+	C+; P+
K14	+	+	±	C-low; P-high
SMA	+	+	+	C-low; P-high
CD10	+	+	+	C+; P+

+: all stained.

±: partial stained.

−: negative.

C: center; P: peripheral.

**Table 2:** Basic information of necropsied marmosets used in this study.

Sample ID	Age (yr)	BW (g)	Necropsy reason	Health status	Parity	Ovarian Evidence of Cycling
30778	2.6	358	Colony management	Moderate colitis and enteritis	Virgin	Cycling
30435	3.3	319	Colony management	Glomerulonephritis, glycogenosis	Virgin	Cycling
30360	5.5	198	Health reasons	Moderate colon hyperplasia	Parous	Cycling
30361	5.7	392	Colony management	Mild heart fibrosis, glycogenosis	Unknown	Cycling
29022	7.1	348	Colony management	Mild nephritis, mild colitis	Nulliparous	Cycling
28558	8.3	277	Health reasons	Enteritis, nephritis	Nulliparous	Cycling
26238	8.5	297	Colony management	Cardiac fibrosis	Parous	Cycling
25383	8.6	479	*Control animal	Moderate glomerulonephritis	Nulliparous	Cycling
26845	10.4	344	*Control animal	Lymphosarcoma, moderate cholycystitis, moderate colitis	Nulliparous	Cycling
27561	10.6	436	*Control animal	Moderate glomerulonephritis, adenomyosis	Nulliparous	Cycling

^*^No treatment control animals used for a separate study.
