# Supplementary material for: Characterization of mammary epithelial stem/progenitor cells and their changes with aging in common marmosets
Source: Sci Rep. 2016 Aug 25;6:32190. doi: 10.1038/srep32190 (PMC4997341; doi:10.1038/srep32190)

Characterization of mammary epithelial stem/progenitor cells and their changes with aging in common marmosets

Anqi Wu,[1,2] Qiaoxiang Dong[1,2]*, Hui Gao,[1,2] Yuanshuo Shi,[1,2] Yuanhong Chen,[1,2] Fuchuang Zhang,[1,2] Abhik Bandyopadhyay,[2,3] Danhan Wang,[1] Karla M. Gorena,[4] Changjiang Huang,[1,]* Suzette Tardif,[2,5,6] Peter W. Nathanielsz,[7] Lu-Zhe Sun,[8,2,3]*

[1]Institute of Environmental Safety and Human Health, School of Laboratory Medicine and Life Science, Wenzhou Medical University, University Town, Wenzhou 325035, China

[2]Department of Cellular & Structural Biology, University of Texas Health Science Center, San Antonio, Texas 78229, United States

[3]Cancer Therapy and Research Center, University of Texas Health Science Center, San Antonio, Texas 78229, United States

[4]Flow Cytometry Facility, University of Texas Health Science Center, San Antonio, Texas 78229, United States

[5]Barshop Institute for Longevity and Aging Studies, University of Texas Health Science Center, San Antonio, Texas 78229, United States

[6] Southwest National Primate Research Center, Texas Biomedical Research Institute, San Antonio, TX, 78245, United States

[7]Center for Pregnancy and Newborn Research, University of Texas Health Science Center, San Antonio, Texas 78229

[8]The Second Affiliated Hospital, Wenzhou Medical University, Wenzhou 325035, China

*Corresponding authors: dqxdong@163.com or cjhuang5711@163.com or sunl@uthscsa.edu

Supplemental information

Fig. S1 Whole mount staining of the mammary gland from a 2-year old female marmoset. Scale bar, 200 µm

Fig. S2 *In vitro* type I and type II luminal-like colonies stained for luminal markers of K8 and K18 and basal markers of K14, SMA, K5, and CD10. Scale bars, 100 µm

Fig. S3 *In vitro* type III myoepithelial-like colony stained for luminal markers of K8 and K18 and basal markers of K14, SMA, K5, and CD10. Scale bars, 100 µm

Fig. S4 The distribution of different types of colonies in CD49f low vs. high cells in each animal, which was coded with a numerical number and age shown on the top of each pie chart. The number (N) of colonies formed for each animal was derived from a sample of 10,000 cells

Fig. S5 *In vitro* luminal-like colonies formed by cells dissociated from 3D organoids and stained for luminal markers of K8 and ESA (*a.k.a* EpCAM), and basal markers of K14, K5, SMA, and CD10. Scale bars, 100 µm

Fig. S6 *In vitro* myoepithelial-like colonies formed by cells dissociated from 3D organoids and stained for luminal markers of K8 and ESA (*a.k.a* EpCAM), and basal markers of K14, K5, SMA and CD10. Scale bars, 100 µm

Fig. S7 Quantification of colonies formed by cells dissociated from collagen gels harvested 4 weeks post transplantation.

Fig. S8 Representative images showing *in vitro* luminal-like colonies (C1, C2), myoepithelial-like colony (C3), and luminal and myoepithelial mixed colony (Mixed) derived from mammary cells isolated from a 14.8-year old female baboon, and the distribution of different colonies in unsorted cells, sorted CD49f negative and positive cells, and total sorted cells combining the CD49f negative and positive cells. Scale bars,

100 μm

Fig. S9 K8 and K14 staining of *in vitro* luminal-like colonies (C1, C2), myoepithelial-like colony (C3), and mixed colony (Mixed) derived from FACS sorted CD49f positive mammary cells of a 14.8-year old female baboon. Scale bars, 100 μm

Wu et al-Fig. S1

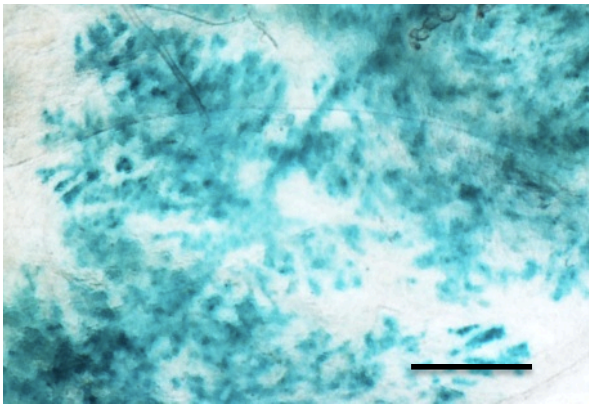

Wu et al-Fig. S2

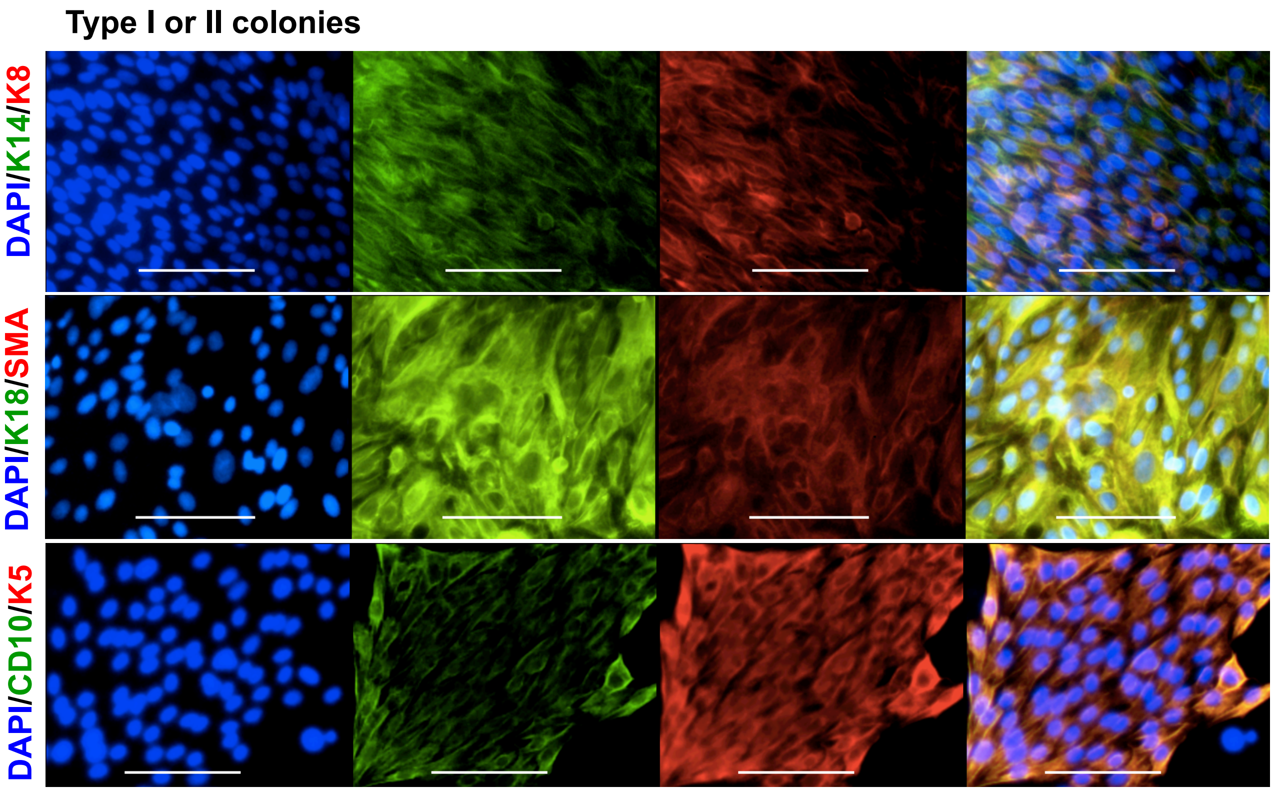

Wu et al-Fig. S3

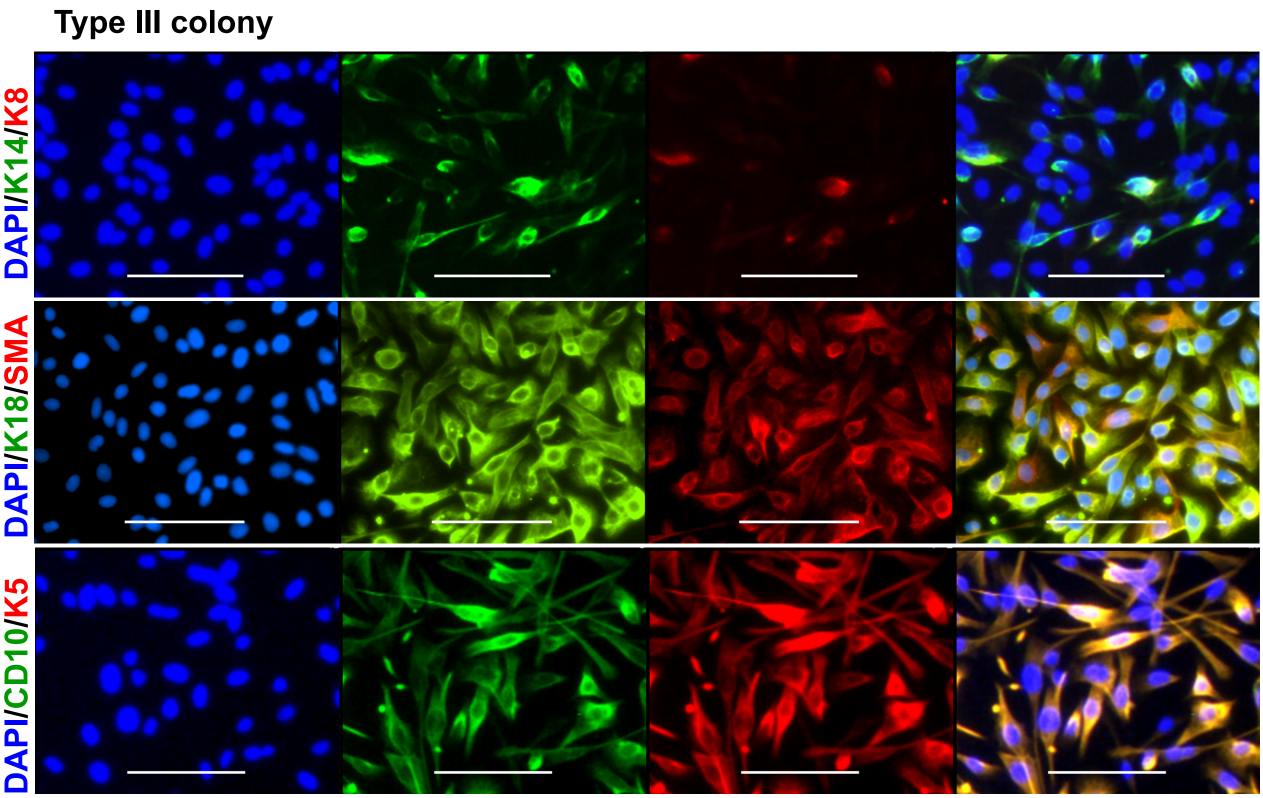

Wu et al-Fig. S4

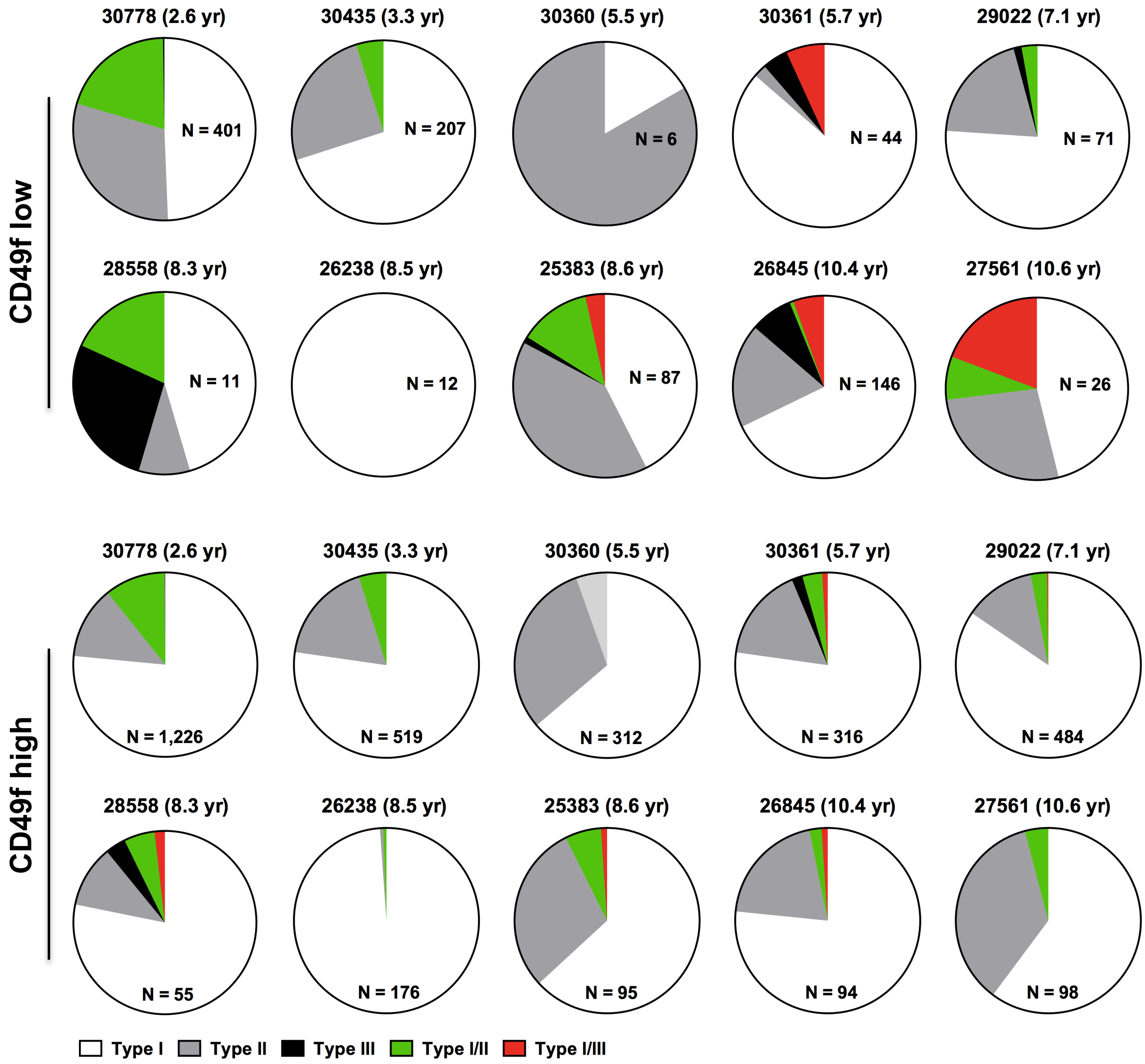

Wu et al-Fig. S5

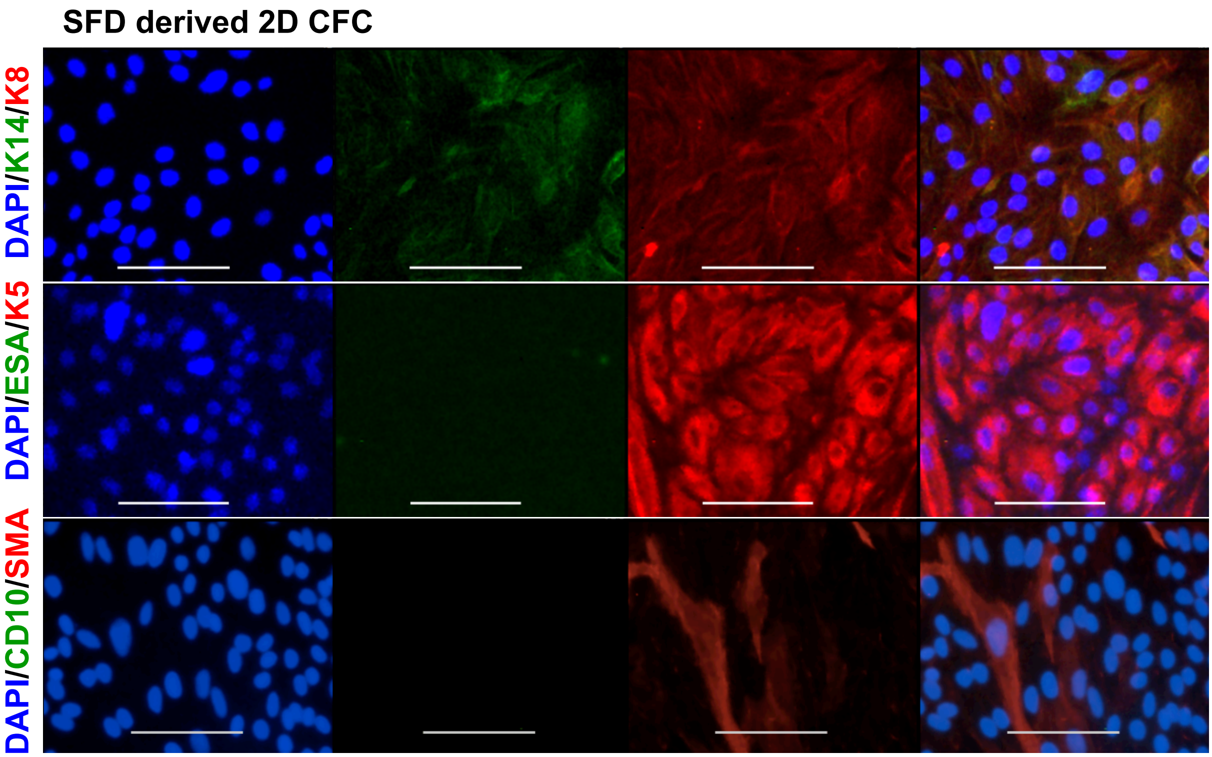

Wu et al-Fig. S6

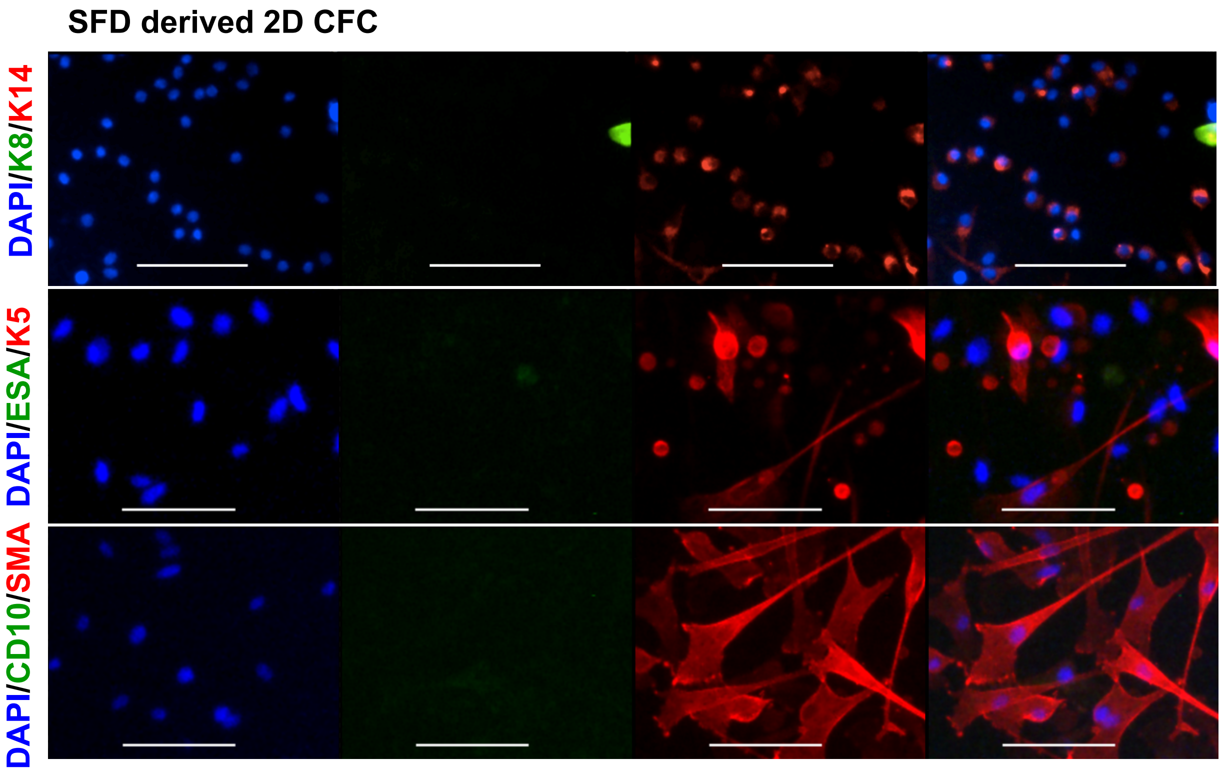

Wu et al-Fig. S7

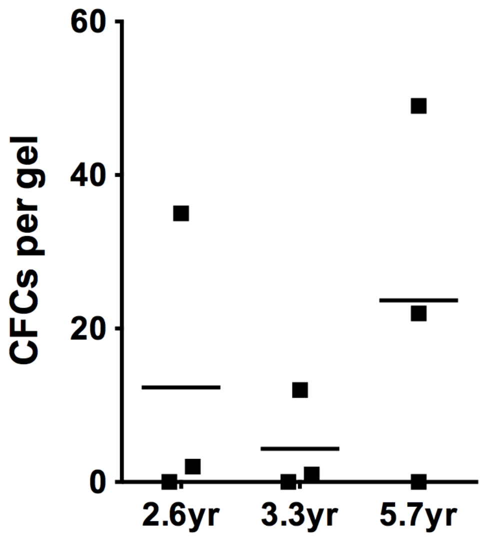

Wu et al-Fig. S8

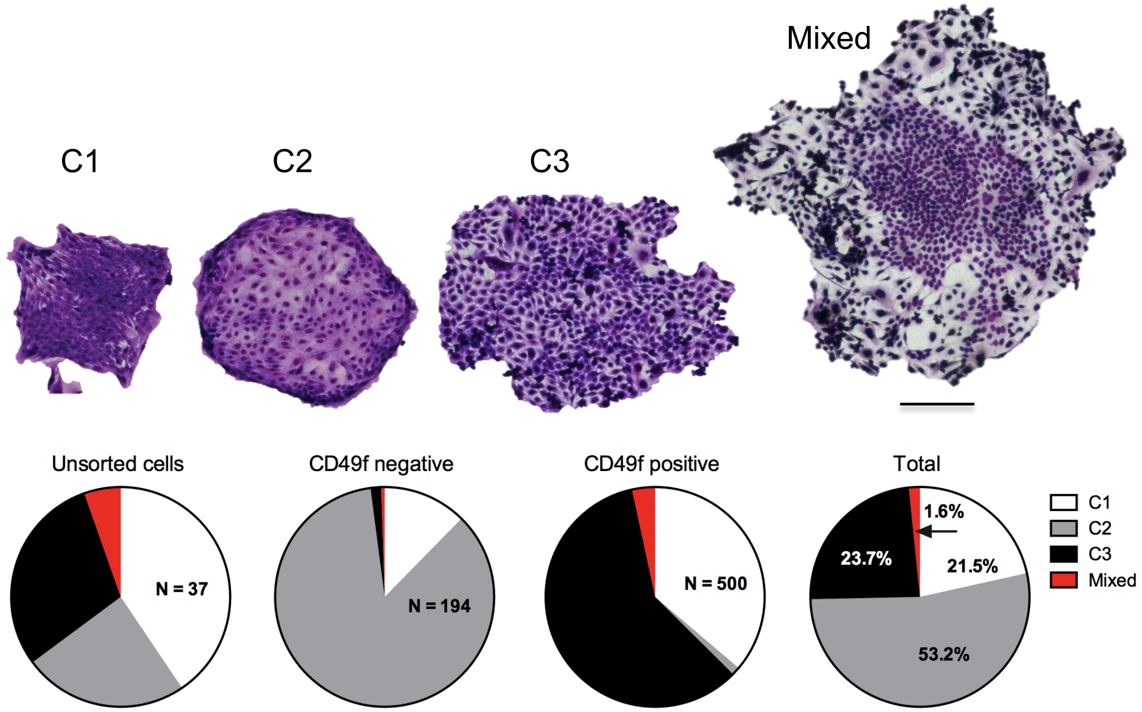

Wu et al-Fig. S9

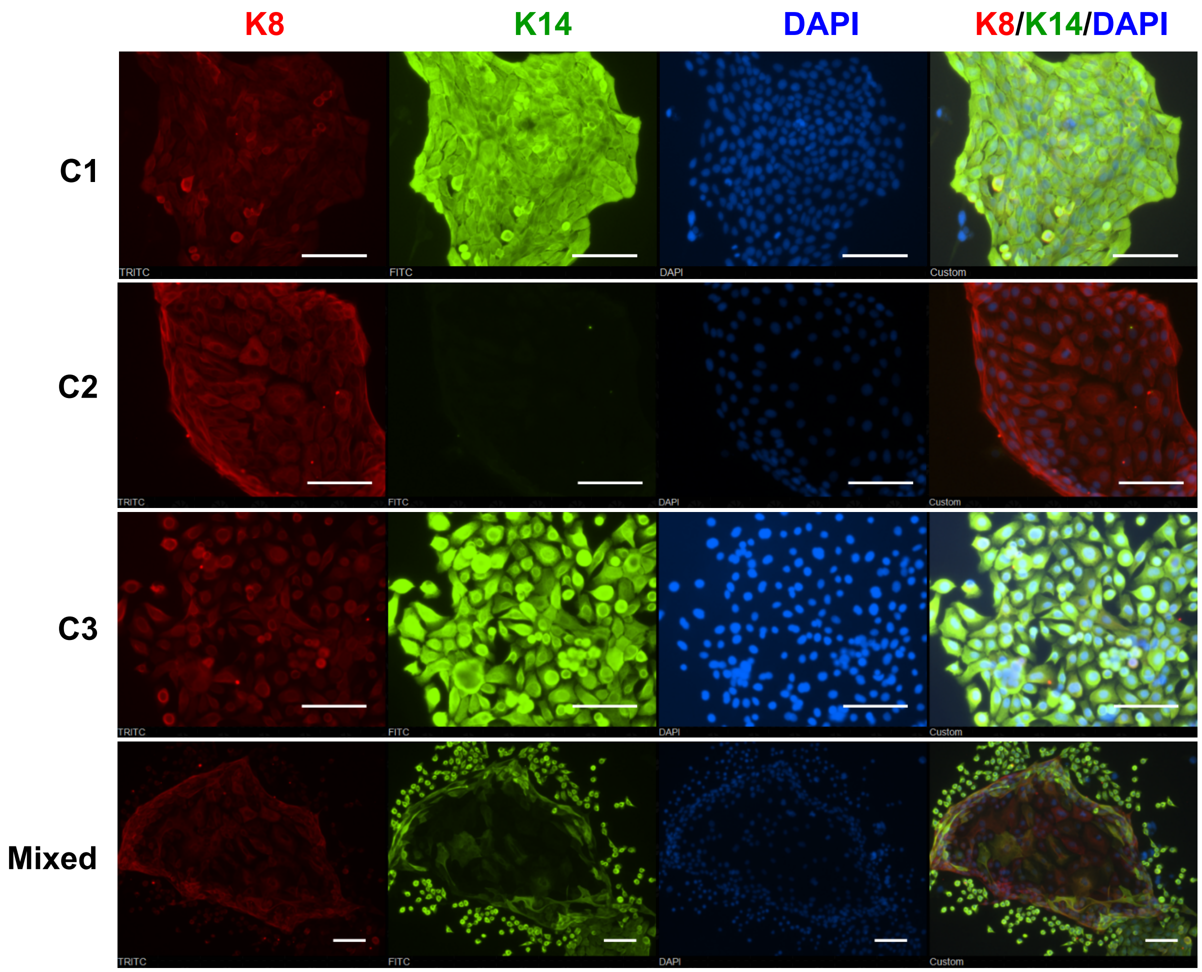

Supplement: Supplementary Information [file srep32190-s1.pdf]
